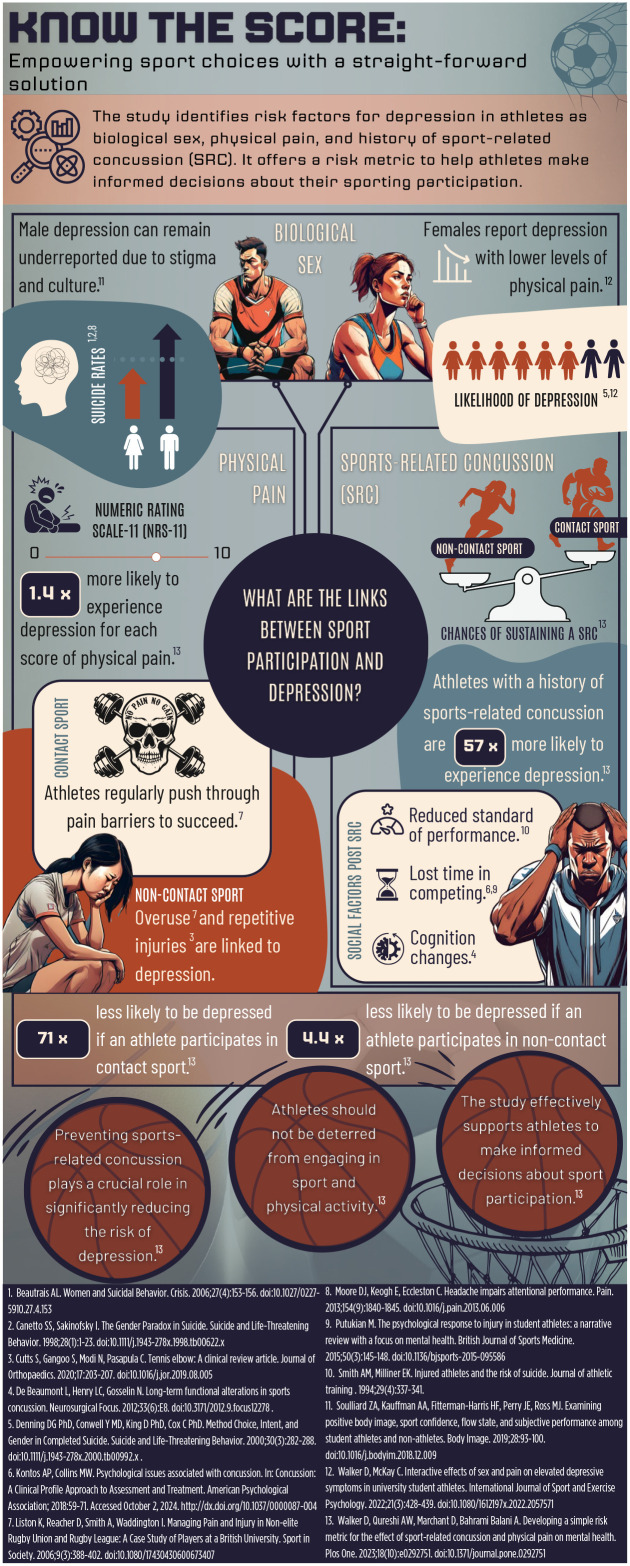# Know the Score: Empowering Sport Choices With a Straightforward Solution

**DOI:** 10.1177/19417381241313374

**Published:** 2025-01-29

**Authors:** Daniel Walker, Jade L. Jukes

**Affiliations:** † University of Bradford, Bradford, UK

**Keywords:** depression, physical pain, sport-related concussion, sportspeople

## Abstract

Risk factors associated with depression in athletes include biological sex, physical pain, and history of sport-related concussion (SRC). However, although there are well-documented benefits of sport and physical activity on mental health, many sportspeople still take the risk of competing in contact sports. Therefore, this infographic, supported by scientific evidence, aims to provide sportspeople with an informed decision on their participation. This infographic can be used by sports clubs or governing bodies to illustrate the risk that SRC has on the mental health of sportspeople. Likewise, it highlights the elevated risk of being in physical pain and being a female sportsperson. Therefore, this infographic provides a simple message to enhance the decision-making process of sportspeople, ensuring they are making a better-informed choice of their sporting participation and making their own cost/reward judgment.

The authors report no potential conflicts of interest in the development and publication of this article.

## KNOW THE SCORE:

### Empowering sport choices with a straight-forward solution

The study identifies risk factors for depression in athletes as biological sex, physical pain, and history of sport-related concussion (SRC). It offers a risk metric to help athletes make informed decisions about their sporting participation.

#### BIOLOGICAL SEX

Male depression can remain underreported due to stigma and culture.[11]

Females report depression with lower levels of physical pain.[12]

SUICIDE RATES [1,2,8]

LIKELIHOOD OF DEPRESSION [5,12]

#### WHAT ARE THE LINKS BETWEEN SPORT PARTICIPATION AND DEPRESSION?

#### PHYSICAL PAIN

NUMERIC RATING SCALE-11 (NRS-11)

0 — 10

**1.4 x** more likely to experience depression for each score of physical pain.[13]

CONTACT SPORT

Athletes regularly push through pain barriers to succeed.[7]

NON-CONTACT SPORT

Overuse[7] and repetitive injuries[3] are linked to depression.

#### SPORTS-RELATED CONCUSSION (SRC)

NON-CONTACT SPORT

CONTACT SPORT

CHANCES OF SUSTAINING A SRC[13]

Athletes with a history of sports-related concussion are **57 x** more likely to experience depression.[13]

SOCIAL FACTORS POST SRC

- Reduced standard of performance.[10]
- Lost time in competing.[6,9]
- Cognition changes.[4]

**71 x** less likely to be depressed if an athlete participates in contact sport.[13]

**4.4 x** less likely to be depressed if an athlete participates in non-contact sport.[13]

Preventing sports-related concussion plays a crucial role in significantly reducing the risk of depression.[13]

Athletes should not be deterred from engaging in sport and physical activity.[13]

The study effectively supports athletes to make informed decisions about sport participation.[13]

1. Beautrais AL. Women and Suicidal Behavior. Crisis. 2006;27(4):153-156. doi:10.1027/0227-5910.27.4.153
2. Canetto SS, Sakinofsky I. The Gender Paradox in Suicide. Suicide and Life-Threatening Behavior. 1998;28(1):1-23. doi:10.1111/j.1943-278x.1998.tb00622.x
3. Cutts S, Gangoo S, Modi N, Pasapula C. Tennis elbow: A clinical review article. Journal of Orthopaedics. 2020;17:203-207. doi:10.1016/j.jor.2019.08.005
4. De Beaumont L, Henry LC, Gosselin N. Long-term functional alterations in sports concussion. Neurosurgical Focus. 2012;33(6):E8. doi:10.3171/2012.9.focus12278 .
5. Denning DG PhD, Conwell Y MD, King D PhD, Cox C PhD. Method Choice, Intent, and Gender in Completed Suicide. Suicide and Life-Threatening Behavior. 2000;30(3):282-288. doi:10.1111/j.1943-278x.2000.tb00992.x .
6. Kontos AP, Collins MW. Psychological issues associated with concussion. In: Concussion: A Clinical Profile Approach to Assessment and Treatment. American Psychological Association; 2018:59-71. Accessed October 2, 2024. http://dx.doi.org/10.1037/0000087-004
7. Liston K, Reacher D, Smith A, Waddington I. Managing Pain and Injury in Non-elite Rugby Union and Rugby League: A Case Study of Players at a British University. Sport in Society. 2006;9(3):388-402. doi:10.1080/17430430600673407
8. Moore DJ, Keogh E, Eccleston C. Headache impairs attentional performance. Pain. 2013;154(9):1840-1845. doi:10.1016/j.pain.2013.06.006
9. Putukian M. The psychological response to injury in student athletes: a narrative review with a focus on mental health. British Journal of Sports Medicine. 2015;50(3):145-148. doi:10.1136/bjsports-2015-095586
10. Smith AM, Milliner EK. Injured athletes and the risk of suicide. Journal of athletic training . 1994;29(4):337-341.
11. Soulliard ZA, Kauffman AA, Fitterman-Harris HF, Perry JE, Ross MJ. Examining positive body image, sport confidence, flow state, and subjective performance among student athletes and non-athletes. Body Image. 2019;28:93-100. doi:10.1016/j.bodyim.2018.12.009
12. Walker D, McKay C. Interactive effects of sex and pain on elevated depressive symptoms in university student athletes. International Journal of Sport and Exercise Psychology. 2022;21(3):428-439. doi:10.1080/1612197x.2022.2057571
13. Walker D, Qureshi AW, Marchant D, Bahrami Balani A. Developing a simple risk metric for the effect of sport-related concussion and physical pain on mental health. Plos One. 2023;18(10):e0292751. doi:10.1371/journal.pone.0292751

### ORCID ID

Daniel Walker https://orcid.org/0000-0002-9369-6953
